# 1-Heptyl-1,3,6,8-tetraaza­tricyclo­[4.3.1.1^3,8^]undecan-1-ium iodide

**DOI:** 10.1107/S1600536811036403

**Published:** 2011-09-14

**Authors:** Augusto Rivera, John Sadat-Bernal, Jaime Ríos-Motta, Karla Fejfarová, Michal Dušek

**Affiliations:** a Departamento de Química, Universidad Nacional de Colombia, Ciudad Universitaria, Bogotá, Colombia; b Institute of Physics ASCR, v.v.i., Na Slovance 2, 182 21 Praha 8, Czech Republic

## Abstract

The title compound C_14_H_29_N_4_^+^·I^−^ salt, was obtained by the reaction of cage adamanzane-type aminal 1,3,6,8-tetra­aza­tricyclo­[4.3.1.1^3,8^]undecane with heptyl iodide. In the cation, the bond lengths and angles are within normal ranges, except for one N—C(ring) bond distance of 1.542 (3) Å, which is unexpectedly long compared with related compounds. In the crystal, ions are linked through C—H⋯I hydrogen bonds. The crystal studied was a non-merohedral twin with a minor twin domain of 6.56 (5)%.

## Related literature

For the preparation of the title compound, see: Rivera *et al.* (2011 ▶). For synthetic applications of quaternary ammonium salts, see: Starks (1971 ▶). For related structures, see: Betz & Klüfers (2007 ▶); Lee *et al.* (2011 ▶).
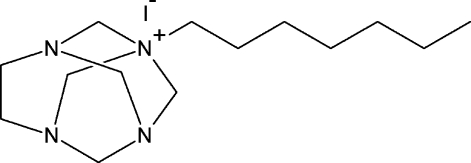


## Experimental

### 

#### Crystal data

C_14_H_29_N_4_^+^·I^−^
*M* *_r_* = 380.3
Monoclinic, 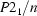

*a* = 8.8325 (2) Å
*b* = 15.3276 (3) Å
*c* = 12.4792 (2) Å
β = 100.072 (2)°
*V* = 1663.41 (6) Å^3^
*Z* = 4
Mo *K*α radiation
μ = 1.92 mm^−1^
*T* = 160 K
0.31 × 0.24 × 0.16 mm

#### Data collection

Agilent Xcalibur diffractometer with Atlas (Gemini ultra Cu) detector
Absorption correction: multi-scan (*CrysAlis PRO*; Agilent, 2010 ▶) *T*_min_ = 0.871, *T*_max_ = 1
22619 measured reflections
4183 independent reflections
3517 reflections with *I* > 3σ(*I*)
*R*_int_ = 0.031

#### Refinement

*R*[*F* ^2^ > 2σ(*F* ^2^)] = 0.027
*wR*(*F* ^2^) = 0.065
*S* = 1.61
4183 reflections
173 parameters
H-atom parameters constrained
Δρ_max_ = 0.53 e Å^−3^
Δρ_min_ = −0.48 e Å^−3^

### 

Data collection: *CrysAlis PRO* (Agilent, 2010 ▶); cell refinement: *CrysAlis PRO*; data reduction: *CrysAlis PRO*; program(s) used to solve structure: *SIR2002* (Burla *et al.*, 2003 ▶); program(s) used to refine structure: *JANA2006* (Petříček *et al.*, 2006 ▶); molecular graphics: Diamond (Brandenburg & Putz, 2005 ▶); software used to prepare material for publication: *JANA2006*.

## Supplementary Material

Crystal structure: contains datablock(s) global, I. DOI: 10.1107/S1600536811036403/bx2371sup1.cif

Structure factors: contains datablock(s) I. DOI: 10.1107/S1600536811036403/bx2371Isup2.hkl

Supplementary material file. DOI: 10.1107/S1600536811036403/bx2371Isup3.cml

Additional supplementary materials:  crystallographic information; 3D view; checkCIF report

## Figures and Tables

**Table 1:** Hydrogen-bond geometry (Å, °)

*D*—H⋯*A*	*D*—H	H⋯*A*	*D*⋯*A*	*D*—H⋯*A*
C2—H2*a*⋯I1^i^	0.96	2.94	3.858 (2)	161

Symmetry code: (i) 
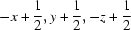
.

**Fig. 1.**
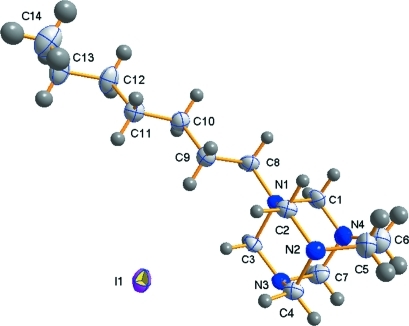
A view of (I) with the numbering scheme. Displacement ellipsoids are drawn at the 50% probability level.

**Fig. 2.**
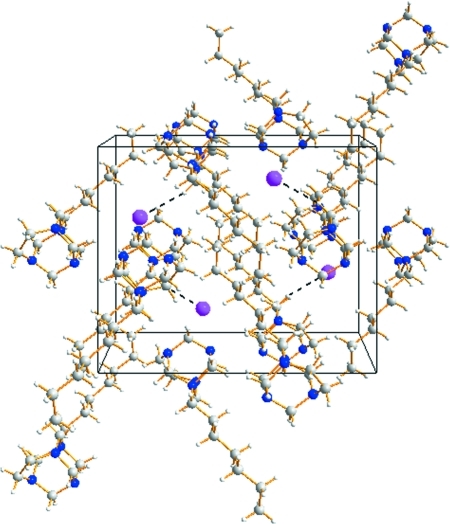
Crystal packing of the title compound view along a axis.

## supplementary crystallographic information

### Comment

Quaternary ammonium salts are used as phase transfer catalysts for a wide range of organic reactions involving immiscible solvent systems (Starks, 1971). Therefore, we have decided to synthesize a new series of new *N*-alkylated quaternary ammonium salts, based on the Menschutkin reaction (Rivera *et al.*, 2011) of 1,3,6,8-tetraazatricyclo[4.3.1.1^3,8^]undecane with an alkyl halide. In the present work, the structure of a new compound, 1-heptyl-1,3,6,8-tretraazatricyclo[4.3.1.1^3,8^]undeca-1-ium iodide, is described.

The molecular geometry and the atom-numbering scheme of (I) are shown in Fig. 1. The asymetric unit of title molecule, C_14_H_29_N_4_^+^.I^-^, contains a 1-heptyl-1,3,6,8-tretraazatricyclo[4.3.1.1^3,8^]undeca-1-ium cation and one iodide anion. Bond lenghts and angles in the title compound are normal, however the bond length N1—C1 [1.542 (3) Å] in the quaternary nitrogen is longer than the corresponding values observed in related structure [1.527 (3) Å] (Betz & Klüfers, 2007). In the cation, the torsion angle on the ethylene bridge is slightly distorted from the exact *D_2_**_d_* symmetry [N2—C5—C6—N4 torsion angle = 7.2 (4)°]. In the crystal, ions are linked by C—H···I hydrogen bonds (Figure 2), which is shorter (Table 1) than the corresponding contacts in related structure (Lee, *et al.*, 2011).The main conformational feature is that the torsion angles in the heptyl chain are further removed from the ideal *all-trans* conformation, notably in C11—C12—C13—C14 fragment, which differ in the relative orientations [C—C—C—C torsion angle = 67.8 (3)°].

### Experimental

The synthetic method has been described earlier (Rivera *et al.*, 2011), except that heptyl idodide was used as alkylating agent. Single crystals suitable for X-ray analysis were obtained by crystallization from methanol solution. *M*.p. = 409–410 K. MS (ESI^+^): *m/z* 253.2441 [C_7_H_14_N_4_^+^C_7_H_15_].

### Refinement

Hydrogen atoms were placed to ideal positions and refined as riding with C–H distance 0.96 Å. The methyl H atoms were allowed to rotate freely about the adjacent C—C bonds. The isotropic atomic displacement parameters of hydrogen atoms were set to 1.2 (CH_2_) or 1.5 (CH_3_) times *U*_eq_ of the parent atom.

### Crystal data

**Table:**

C_14_H_29_N_4_^+^·I^−^	*F*(000) = 776
*M**_r_* = 380.3	*D*_x_ = 1.518 Mg m^−^^3^
Monoclinic, *P*2_1_/*n*	Mo *K*α radiation, λ = 0.7107 Å
Hall symbol: -P 2yn	Cell parameters from 12607 reflections
*a* = 8.8325 (2) Å	θ = 2.9–29.2°
*b* = 15.3276 (3) Å	µ = 1.92 mm^−^^1^
*c* = 12.4792 (2) Å	*T* = 160 K
β = 100.072 (2)°	Irregular shape, colourless
*V* = 1663.41 (6) Å^3^	0.31 × 0.24 × 0.16 mm
*Z* = 4	

### Data collection

**Table:**

Agilent Xcalibur diffractometer with Atlas (Gemini ultra Cu) detector	4183 independent reflections
Radiation source: Enhance (Mo) X-ray Source	3517 reflections with *I* > 3σ(*I*)
graphite	*R*_int_ = 0.031
Detector resolution: 10.3784 pixels mm^-1^	θ_max_ = 29.3°, θ_min_ = 2.9°
Rotation method data acquisition using ω scans	*h* = −11→12
Absorption correction: multi-scan (*CrysAlis PRO*; Agilent, 2010)	*k* = −20→19
*T*_min_ = 0.871, *T*_max_ = 1	*l* = −16→16
22619 measured reflections	

### Refinement

**Table:**

Refinement on *F*^2^	116 constraints
*R*[*F*^2^ > 2σ(*F*^2^)] = 0.027	H-atom parameters constrained
*wR*(*F*^2^) = 0.065	Weighting scheme based on measured s.u.'s *w* = 1/(σ^2^(*I*) + 0.0004*I*^2^)
*S* = 1.61	(Δ/σ)_max_ = 0.016
4183 reflections	Δρ_max_ = 0.53 e Å^−^^3^
173 parameters	Δρ_min_ = −0.48 e Å^−^^3^
0 restraints	

### Special details

**Table:**

Refinement. The refinement was carried out against all reflections. The conventional *R*-factor is always based on *F*. The goodness of fit as well as the weighted *R*-factor are based on *F* and *F*^2^ for refinement carried out on *F* and *F*^2^, respectively. The threshold expression is used only for calculating *R*-factors *etc*. and it is not relevant to the choice of reflections for refinement.The crystal studied was a non-merohedral twin with a minor twin domain of 6.56 (5)%. The overlaps of reflection between the twin domains were calculated by Jana2006 software using the twinning matrix and user- defined treshold 0.15 degs for full overlap. Due to no support for twinning in the official CIF dictionary the twinning matrix has been saved in the CIF file using special _jana_cell_twin_matrix keyword.The program used for refinement, Jana2006, uses the weighting scheme based on the experimental expectations, see _refine_ls_weighting_details, that does not force *S* to be one. Therefore the values of *S* are usually larger than the ones from the *SHELX* program.

### Fractional atomic coordinates and isotropic or equivalent isotropic displacement parameters (Å^2^)

**Table:**

	*x*	*y*	*z*	*U*_iso_*/*U*_eq_	
I1	0.255234 (19)	0.362795 (11)	0.161798 (13)	0.03611 (6)	
N1	0.1652 (2)	0.66563 (12)	0.09078 (15)	0.0265 (6)	
N2	0.4475 (2)	0.65925 (13)	0.13273 (16)	0.0299 (6)	
N3	0.3065 (2)	0.60189 (13)	−0.03990 (16)	0.0314 (6)	
C1	0.1645 (3)	0.75253 (15)	0.0285 (2)	0.0343 (8)	
C2	0.3103 (3)	0.65513 (15)	0.17770 (19)	0.0285 (7)	
C3	0.1707 (3)	0.59276 (16)	0.00795 (18)	0.0304 (7)	
C4	0.4444 (3)	0.59596 (16)	0.04572 (19)	0.0321 (8)	
C5	0.5050 (4)	0.74388 (18)	0.1150 (3)	0.0570 (12)	
C6	0.4250 (4)	0.7963 (2)	0.0231 (3)	0.0625 (13)	
N4	0.2865 (3)	0.76063 (14)	−0.03149 (18)	0.0417 (8)	
C7	0.2995 (3)	0.68432 (18)	−0.0992 (2)	0.0406 (9)	
C8	0.0209 (3)	0.66349 (16)	0.13859 (19)	0.0318 (8)	
C9	0.0031 (3)	0.58651 (17)	0.2112 (2)	0.0362 (8)	
C10	−0.1575 (3)	0.58205 (17)	0.2378 (2)	0.0361 (8)	
C11	−0.1784 (3)	0.50740 (18)	0.3136 (2)	0.0376 (8)	
C12	−0.3416 (3)	0.4983 (2)	0.3350 (2)	0.0490 (10)	
C13	−0.3676 (3)	0.4201 (2)	0.4036 (2)	0.0543 (11)	
C14	−0.2874 (4)	0.4242 (2)	0.5197 (3)	0.0644 (13)	
H1a	0.067925	0.759245	−0.019773	0.0411*	
H1b	0.168363	0.800294	0.078623	0.0411*	
H2a	0.312341	0.699798	0.231869	0.0343*	
H2b	0.30621	0.600427	0.214549	0.0343*	
H3a	0.172331	0.537227	0.043705	0.0365*	
H3b	0.081192	0.596113	−0.047999	0.0365*	
H4a	0.452199	0.538155	0.075802	0.0385*	
H4b	0.534905	0.602573	0.013598	0.0385*	
H5a	0.612201	0.739939	0.110647	0.0684*	
H5b	0.514452	0.777325	0.180856	0.0684*	
H6a	0.406609	0.853891	0.048084	0.075*	
H6b	0.492883	0.806321	−0.02793	0.075*	
H7a	0.389014	0.689961	−0.132646	0.0488*	
H7b	0.214378	0.683107	−0.158738	0.0488*	
H8a	0.011991	0.716719	0.177567	0.0381*	
H8b	−0.066861	0.666772	0.081196	0.0381*	
H9a	0.076657	0.591093	0.277414	0.0435*	
H9b	0.024842	0.533548	0.175699	0.0435*	
H10a	−0.181244	0.636201	0.269808	0.0433*	
H10b	−0.230927	0.576603	0.171639	0.0433*	
H11a	−0.147264	0.453761	0.284218	0.0451*	
H11b	−0.109258	0.514772	0.38146	0.0451*	
H12a	−0.411644	0.496068	0.266936	0.0588*	
H12b	−0.370287	0.550579	0.368846	0.0588*	
H13a	−0.475937	0.412162	0.40143	0.0651*	
H13b	−0.337479	0.367937	0.370327	0.0651*	
H14a	−0.178217	0.42272	0.52221	0.0966*	
H14b	−0.317757	0.375148	0.558892	0.0966*	
H14c	−0.314979	0.477258	0.552303	0.0966*	

### Atomic displacement parameters (Å^2^)

**Table:**

	*U*^11^	*U*^22^	*U*^33^	*U*^12^	*U*^13^	*U*^23^
I1	0.03178 (10)	0.03586 (11)	0.03959 (11)	−0.00013 (7)	0.00324 (7)	0.01196 (7)
N1	0.0256 (10)	0.0264 (10)	0.0269 (10)	0.0031 (8)	0.0033 (8)	−0.0015 (8)
N2	0.0258 (10)	0.0313 (11)	0.0314 (11)	0.0012 (8)	0.0019 (9)	−0.0028 (9)
N3	0.0340 (11)	0.0326 (11)	0.0276 (10)	0.0013 (9)	0.0050 (9)	−0.0032 (9)
C1	0.0380 (14)	0.0266 (13)	0.0377 (14)	0.0063 (11)	0.0052 (12)	0.0061 (11)
C2	0.0283 (12)	0.0314 (13)	0.0245 (11)	0.0047 (10)	0.0006 (10)	−0.0018 (9)
C3	0.0320 (13)	0.0281 (12)	0.0297 (12)	−0.0004 (10)	0.0013 (10)	−0.0054 (10)
C4	0.0310 (13)	0.0315 (13)	0.0342 (13)	0.0049 (11)	0.0068 (11)	−0.0007 (11)
C5	0.0535 (19)	0.0419 (17)	0.081 (2)	−0.0115 (15)	0.0258 (18)	−0.0055 (16)
C6	0.056 (2)	0.057 (2)	0.076 (2)	−0.0098 (17)	0.0148 (18)	−0.0051 (18)
N4	0.0476 (14)	0.0321 (12)	0.0487 (13)	0.0000 (10)	0.0170 (11)	0.0071 (10)
C7	0.0456 (16)	0.0481 (16)	0.0287 (13)	0.0058 (13)	0.0076 (12)	0.0075 (12)
C8	0.0272 (12)	0.0331 (13)	0.0348 (13)	0.0050 (10)	0.0049 (11)	−0.0008 (11)
C9	0.0341 (13)	0.0379 (14)	0.0366 (13)	0.0017 (12)	0.0060 (11)	0.0039 (11)
C10	0.0304 (13)	0.0367 (14)	0.0409 (14)	0.0003 (11)	0.0055 (11)	0.0015 (11)
C11	0.0344 (14)	0.0394 (15)	0.0379 (14)	−0.0011 (11)	0.0035 (12)	0.0021 (11)
C12	0.0326 (15)	0.0578 (19)	0.0547 (18)	−0.0084 (13)	0.0026 (13)	0.0159 (15)
C13	0.0458 (18)	0.059 (2)	0.0562 (18)	−0.0162 (15)	0.0040 (15)	0.0130 (16)
C14	0.058 (2)	0.083 (3)	0.0521 (19)	−0.0093 (19)	0.0083 (17)	0.0134 (18)

### Geometric parameters (Å, °)

**Table:**

N1—C1	1.542 (3)	N4—C7	1.459 (4)
N1—C2	1.536 (3)	C7—H7a	0.96
N1—C3	1.528 (3)	C7—H7b	0.96
N1—C8	1.499 (3)	C8—C9	1.512 (4)
N2—C2	1.424 (3)	C8—H8a	0.96
N2—C4	1.453 (3)	C8—H8b	0.96
N2—C5	1.424 (4)	C9—C10	1.514 (4)
N3—C3	1.437 (3)	C9—H9a	0.96
N3—C4	1.476 (3)	C9—H9b	0.96
N3—C7	1.460 (3)	C10—C11	1.517 (4)
C1—N4	1.421 (4)	C10—H10a	0.96
C1—H1a	0.96	C10—H10b	0.96
C1—H1b	0.96	C11—C12	1.517 (4)
C2—H2a	0.96	C11—H11a	0.96
C2—H2b	0.96	C11—H11b	0.96
C3—H3a	0.96	C12—C13	1.515 (4)
C3—H3b	0.96	C12—H12a	0.96
C4—H4a	0.96	C12—H12b	0.96
C4—H4b	0.96	C13—C14	1.498 (4)
C5—C6	1.475 (4)	C13—H13a	0.96
C5—H5a	0.96	C13—H13b	0.96
C5—H5b	0.96	C14—H14a	0.96
C6—N4	1.402 (4)	C14—H14b	0.96
C6—H6a	0.96	C14—H14c	0.96
C6—H6b	0.96	C14—C9^i^	3.829 (4)
			
C1—N1—C2	112.07 (17)	C6—N4—C7	116.3 (2)
C1—N1—C3	106.73 (17)	N3—C7—N4	113.6 (2)
C1—N1—C8	106.94 (18)	N3—C7—H7a	109.4709
C2—N1—C3	106.25 (17)	N3—C7—H7b	109.4704
C2—N1—C8	112.29 (18)	N4—C7—H7a	109.472
C3—N1—C8	112.51 (18)	N4—C7—H7b	109.4716
C2—N2—C4	111.04 (18)	H7a—C7—H7b	105.0122
C2—N2—C5	116.9 (2)	N1—C8—C9	116.1 (2)
C4—N2—C5	116.9 (2)	N1—C8—H8a	109.4708
C3—N3—C4	109.67 (18)	N1—C8—H8b	109.4713
C3—N3—C7	109.3 (2)	C9—C8—H8a	109.4716
C4—N3—C7	112.12 (19)	C9—C8—H8b	109.471
N1—C1—N4	113.9 (2)	H8a—C8—H8b	101.9387
N1—C1—H1a	109.4716	C8—C9—C10	111.4 (2)
N1—C1—H1b	109.4717	C8—C9—H9a	109.4711
N4—C1—H1a	109.4708	C8—C9—H9b	109.4714
N4—C1—H1b	109.4711	C10—C9—H9a	109.4709
H1a—C1—H1b	104.657	C10—C9—H9b	109.4708
N1—C2—N2	112.32 (19)	H9a—C9—H9b	107.4338
N1—C2—H2a	109.4714	C9—C10—C11	113.1 (2)
N1—C2—H2b	109.4711	C9—C10—H10a	109.4709
N2—C2—H2a	109.4715	C9—C10—H10b	109.4711
N2—C2—H2b	109.4706	C11—C10—H10a	109.4712
H2a—C2—H2b	106.4595	C11—C10—H10b	109.4712
N1—C3—N3	109.72 (19)	H10a—C10—H10b	105.6299
N1—C3—H3a	109.4716	C10—C11—C12	113.7 (2)
N1—C3—H3b	109.4707	C10—C11—H11a	109.4711
N3—C3—H3a	109.471	C10—C11—H11b	109.4709
N3—C3—H3b	109.4713	C12—C11—H11a	109.4715
H3a—C3—H3b	109.223	C12—C11—H11b	109.4715
N2—C4—N3	113.9 (2)	H11a—C11—H11b	104.9043
N2—C4—H4a	109.4712	C11—C12—C13	114.5 (2)
N2—C4—H4b	109.4712	C11—C12—H12a	109.471
N3—C4—H4a	109.4716	C11—C12—H12b	109.4716
N3—C4—H4b	109.471	C13—C12—H12a	109.4711
H4a—C4—H4b	104.6525	C13—C12—H12b	109.4712
N2—C5—C6	118.8 (3)	H12a—C12—H12b	103.914
N2—C5—H5a	109.4717	C12—C13—C14	114.9 (3)
N2—C5—H5b	109.4715	C12—C13—H13a	109.4717
C6—C5—H5a	109.4709	C12—C13—H13b	109.4715
C6—C5—H5b	109.4709	C14—C13—H13a	109.4714
H5a—C5—H5b	98.1968	C14—C13—H13b	109.4712
C5—C6—N4	115.1 (3)	H13a—C13—H13b	103.4432
C5—C6—H6a	109.4709	C13—C14—H14a	109.4716
C5—C6—H6b	109.4718	C13—C14—H14b	109.4709
N4—C6—H6a	109.4712	C13—C14—H14c	109.4713
N4—C6—H6b	109.4714	H14a—C14—H14b	109.4705
H6a—C6—H6b	103.2278	H14a—C14—H14c	109.4719
C1—N4—C6	117.1 (2)	H14b—C14—H14c	109.4712
C1—N4—C7	112.3 (2)		
			
C11—C12—C13—C14	67.8 (4)	N2—C5—C6—N4	7.2 (4)

Symmetry codes: (i) −*x*, −*y*+1, −*z*+1.

### Hydrogen-bond geometry (Å, °)

**Table:**

*D*—H···*A*	*D*—H	H···*A*	*D*···*A*	*D*—H···*A*
C2—H2a···I1^ii^	0.96	2.94	3.858 (2)	161

Symmetry codes: (ii) −*x*+1/2, *y*+1/2, −*z*+1/2.
